# Flowering in Persian walnut: patterns of gene expression during flower development

**DOI:** 10.1186/s12870-020-02372-w

**Published:** 2020-04-03

**Authors:** Amin Hassankhah, Majid Rahemi, Hossein Ramshini, Saadat Sarikhani, Kourosh Vahdati

**Affiliations:** 1 Department of Horticulture, College of Aburaihan, University of Tehran, Tehran, Iran; 2 Department of Horticultural Sciences, Faculty of Agriculture, Shiraz University, Shiraz, Iran; 3 Department of Agronomy and Plant Breeding Sciences, College of Aburaihan, University of Tehran, Tehran, Iran; 4 Department of Horticulture, College of Aburaihan, University of Tehran, Tehran, Iran; 5 Department of Horticulture, College of Aburaihan, University of Tehran, Tehran, Iran

**Keywords:** Anthesis, Dormancy, Expression, Flower, *FT*, Gene, Induction, Initiation, Persian walnut, *TFL1*

## Abstract

**Background:**

Flower development and sufficient fruit set are important parameters with respect to walnut yield. Knowledge about flowering genes of fruit trees can help to conduct better molecular breeding programs. Therefore, this study was carried out to investigate the expression pattern of some flowering genes (*FT*, *SOC1*, *CAL*, *LFY* and *TFL1*) in Persian walnut (cv. Chandler) during the growing season and winter dormancy.

**Results:**

The results showed that walnut flower induction and initiation in Shahmirzad, Iran occurred in early June and late September, respectively. After meeting chilling and heat requirement, flower differentiation and anthesis occurred in late-March and mid-April to early-May, respectively. Study of flowering gene expression showed that the expression of the *FT* gene increased in three stages including before breaking of bud dormancy, from late March to late April (coincided with flower differentiation and anthesis) and from late May to mid-June (coincided with flower induction). Like *FT*, the expression of *SOC1* gene increased during flower induction and initiation (mid-May to early-August) as well as flower anthesis (mid-April to early-May). *LFY* and *CAL* genes as floral meristem identity genes are activated by *FT* and *SOC1* genes. In contrast with flowering stimulus genes, *TFL1* showed overexpression during winter dormancy which prevented flowering.

**Conclusion:**

The expression of *FT* gene activated downstream floral meristem identity genes including *SOC1*, *CAL* and *LFY* which consequently led to release bud dormancy as well as flower anthesis and induction. Also, *TFL1* as a flowering inhibitor gene in walnut showed overexpression during the bud dormancy. Chilling accumulation reduced *TFL1* gene expression and increased the expression of flowering genes which ultimately led to overcome dormancy.

## Background

Flowering is considered as an important parameter with respect to crop yield and avoid of late-spring frost [1, 2]. In addition to the genetic network controlling flower development, there are a number of much less studied metabolites and exogenous factors such as chilling requirement that may influence the transduction to flowering as well as flower anthesis [3]. Knowledge about flowering genes of fruit trees is scarce. On the other hand, some aspects of flowering in fruit trees such as juvenile and long generation time are the especial challenges to study flower development genes in fruit trees [1]. In addition, flower induction and initiation of many fruit trees occurs in the previous year and therefore the vegetative growth, flowers, and fruits of the previous year have significant effects on flowering in the current year [4].

In order to understand the flowering phenomenon of perennial plants, physiologists first studied flowering of *Arabidopsis thaliana* as a model plant [5]. The *ABC* model was the primary model in the flower development proposed for *Arabidopsis thaliana* [6] and *Antirrhinum majus* [7]. This model postulates the function of three different classes of gene activities A, B and C which together determine floral organ identity [5, 8]. The *ABC* model has been approved in a wide range of higher plants and many molecular studies largely support this model [9, 10]. The floral meristem identity genes induce the different floral organs including sepals, petals, stamens, and carpels according to the *ABC* model [11].

Previous studies indicated that *FLOWERING LOCUS T* (*FT*) is a key gene in plant flowering especially in response to photoperiod. *FT* as an upstream gene promotes floral meristem identity by activating other flowering genes [12]. In addition to *FT* gene, *SOC1* (*SUPRESSOR OF OVEREXPRESSION OF CONSTANS 1*), *CAL* (*CAULIFLOWER*) and *LFY* (*LEAFY*) are other key genes involved in floral meristem identity and flowering time. The *SOC1* is one of the main genes required for the timely activation of B and C floral organ identity genes [13]. The *CAL* as a MADS-box gene is closely related to *APETALA1* (*AP1*), and encodes proteins involved in the floral meristems formation [14]. Like *CAL, LEAFY* (*LFY*) is a floral meristem identity gene which is an important element of the transition from the vegetative to the reproductive phase [15]. In contrast, *TFL1* is a key gene involved in flowering time and development having reverse function of the above-mentioned genes. In other words, *TFL1* gene represses flowering and has a key role in bud dormancy during the winter [16].

Although, the molecular aspect of flower development in *Arabidopsis* has been widely studied in recent years, the expression pattern of major genes involved in flower development of fruit trees such as walnut are almost obscure. Fruit trees have a long vegetative phase before reproductive phase and the flowering cycle repeats for successive years [17]. In temperate deciduous species, budbreak and flowering is dependent on sufficient winter chilling [18]. Currently, researchers are widely using transcriptomics and other powerful genetic tools based on next generation sequencing to study complex gene networks involved in flowering. These studies provide a comprehensive understanding of the gene network involved in flowering of model and non-model plants [19, 20].

Persian walnut (*Juglans regia* L.) is a major nut crop in the world which originated from old Persia. Iran as one of the main origin and distribution centers of walnut has a significant share of the worldwide walnut industry and is one of the leading countries in the walnut production [21, 22]. Due to limited information on the precise timing of the different flower development stages in walnut as well as the gene network involved in walnut flowering, studying the expression pattern of major genes involved in flowering and its relation to different stages of walnut flower development can enhance our knowledge for future studies. The objective of the current study was to investigate the expression of some flowering genes during walnut flower development and to understand the pattern of expression of these genes at different flower development stages (from flower induction to anthesis) during the growing season and winter dormancy.

## Results

The results showed that walnut flower induction occurred in early June in two consecutive years under the conditions prevailed in Shahmirzad, Iran. At this stage, the buds were deformed from a sharp tip to a flat and dome-shape buds. The buds did not show any significant change during summer. Nevertheless, flower initiation was observed in late-September and the pedicles and vascular bundles were visible by optical microscope. During winter dormancy, walnut flower buds increased in weight and entered maturation. After meeting chilling and heat requirement, the bud development started at the end of March. Flower anthesis of walnut trees (cv. Chandler) occurred from mid-April to early-May under Shahmirzad condition (Additional file 2).

**Fig. 1:**
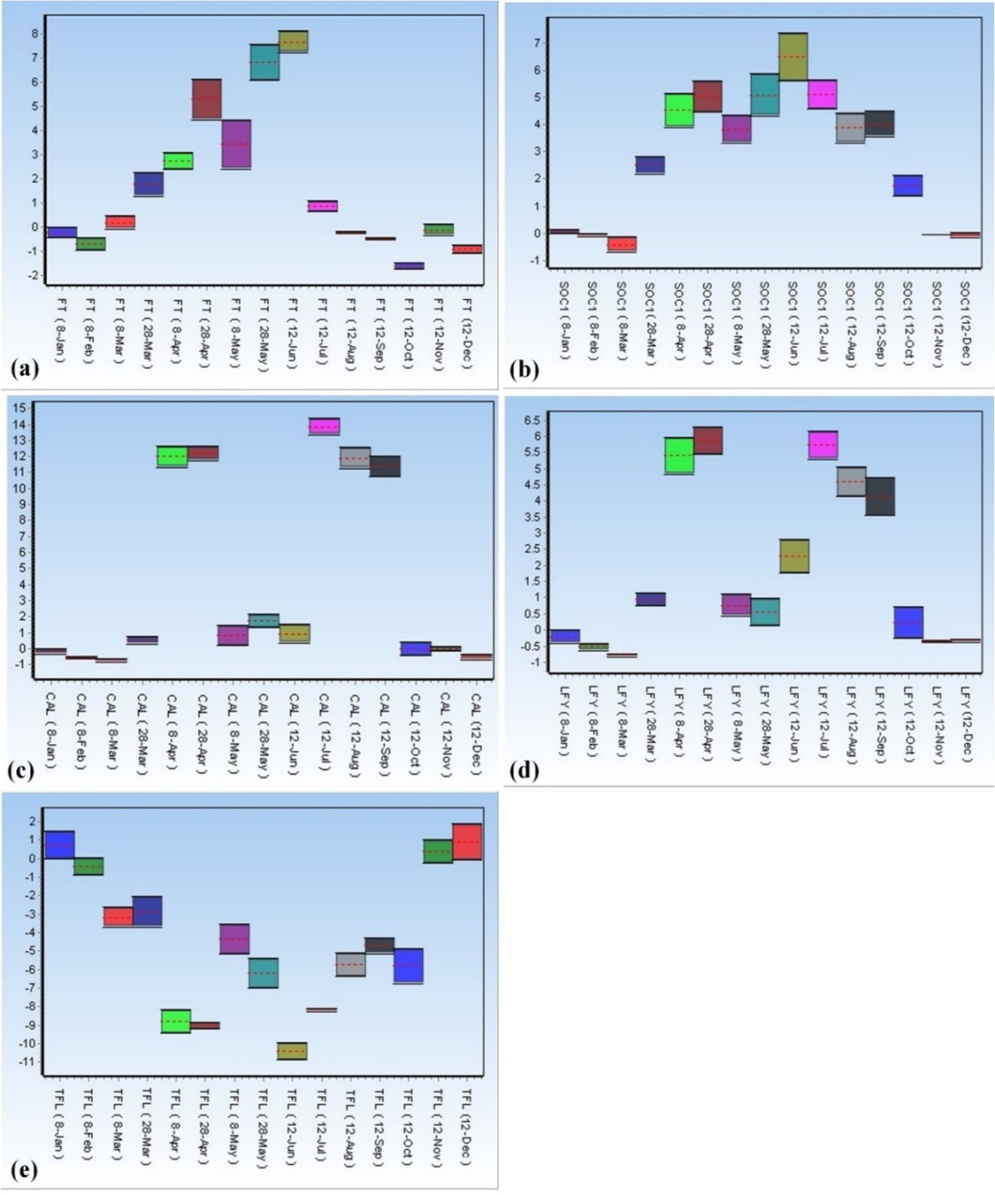
Expression levels of flowering genes in walnut terminal buds (**a**: *FT* gene; **b**: *SOC1* gene; **c**: *CAL* gene; **d**: *LFY* gene; **e**: *TFL1* gene)

There were significant differences among the expression of several genes, i.e. *FT*, *SOC1*, *CAL*, *FLY* and *TFL1*, in walnut buds at different times of the year. Figure 1 and Additional file 3 show the BOXPLOT and fold change diagrams, respectively, pertaining to the studied genes in the walnut buds. Results showed that the expression of the *FT* gene increased in buds from late March. This upward trend lasted until late April (coincided with flower differentiation and anthesis). Then, the *FT* gene exhibited its expression peak in late May to mid-June (coincided with flower induction), and thereafter the expression declined in the buds. From mid-August onwards, this gene was not expressed in the terminal buds of walnuts (Fig. 1a).

With regard to the *SOC1* gene, its expression began late-March (during flower differentiation) and gradually acquired high levels by end of the growth season. The highest level of its expression was recorded in mid-June when the onset of flowering and the induction of flowering were observed in walnuts. The level of its relative expression gradually decreased thereafter, and fell to almost the reference level (8th of June) at the end of the growing season (Fig. 1b).

*CAL* is a floral meristem identity gene that has a very close relationship with the *APETALA1* gene (*AP1*). We observed that the expression of the *CAL* gene increased suddenly from early April. This increase was boosted from mid-April until the start of flowering. Then, a sharp decrease in the expression of the *CAL* gene was observed. A subsequent significant increase in its expression was observed in mid-July until mid-September. Nonetheless, this gene was not expressed from harvest time (early-October) until the start of the next growing season (late-March) (Fig. 1c). The expression analysis of the *LFY* gene generated results that were almost similar to the *CAL* gene. However, the fold change level of *LFY* expression was generally lower than that of the *CAL* gene. Like the *CAL* gene, the expression of the *LFY* gene exhibited two major peaks during the growth season. The first peak was observed at the beginning of the growing season (late-March). The second peak occurred concurrently with the onset of flowering and with the induction of flowering in mid-late summer. From harvest time (early-October) onwards, the expression of *LFY* reached to the reference level (Fig. 1d).

As a protein, *TFL1* is structurally similar to *FT* but with different functions. A decrease in the presence of *TFL1* can accelerate the induction of flowering. Real-time PCR analysis showed that the *TFL1* gene expressed at the end of the walnut growth season and its expression peaked at the beginning of January. The high level of its expression was maintained during the first month of the year and until the beginning of March. Nonetheless, the expression of *TFL1* decreased with the onset of growth season and almost minimized at the time of flowering. Then, its expression increased in May, but again stopped at the beginning of July, parallel to the onset of flower induction (Fig. 1e).

**Fig. 2:**
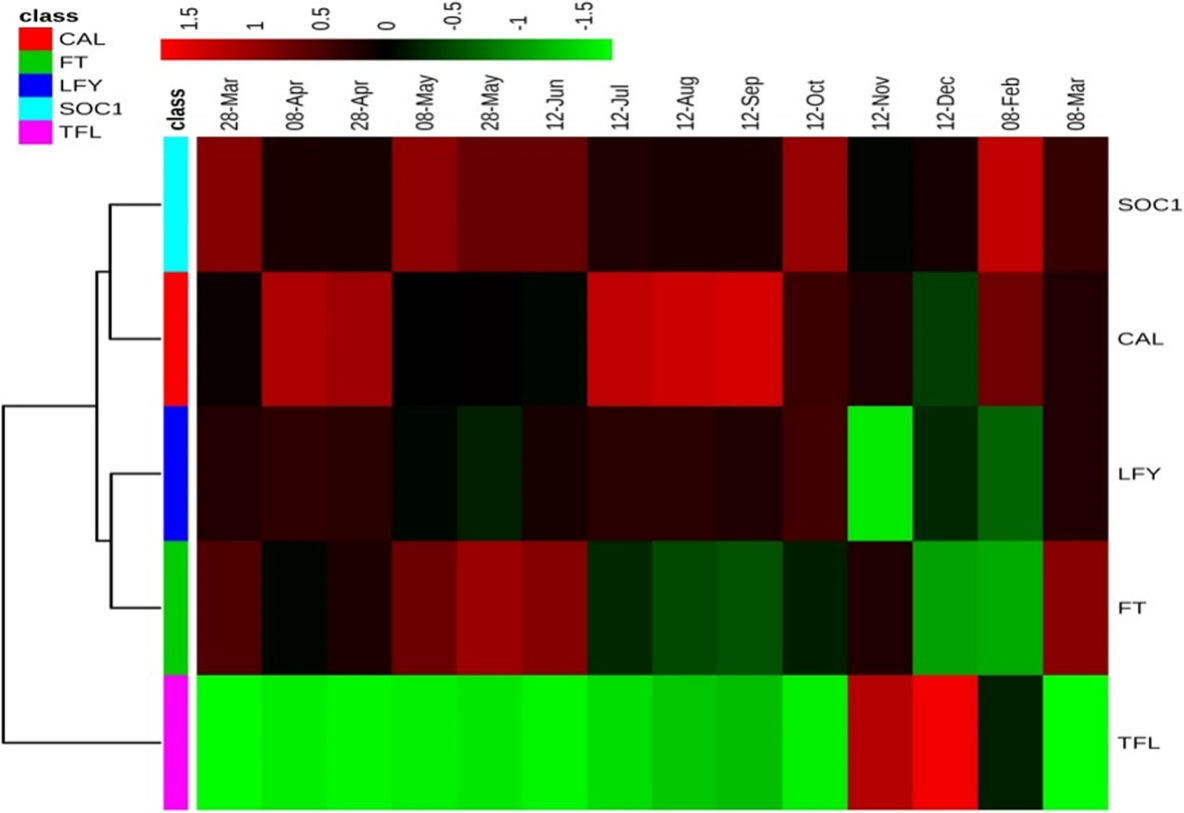
Heatmap cluster of the studied gene expression involved in walnut flowering

**Fig. 3:**
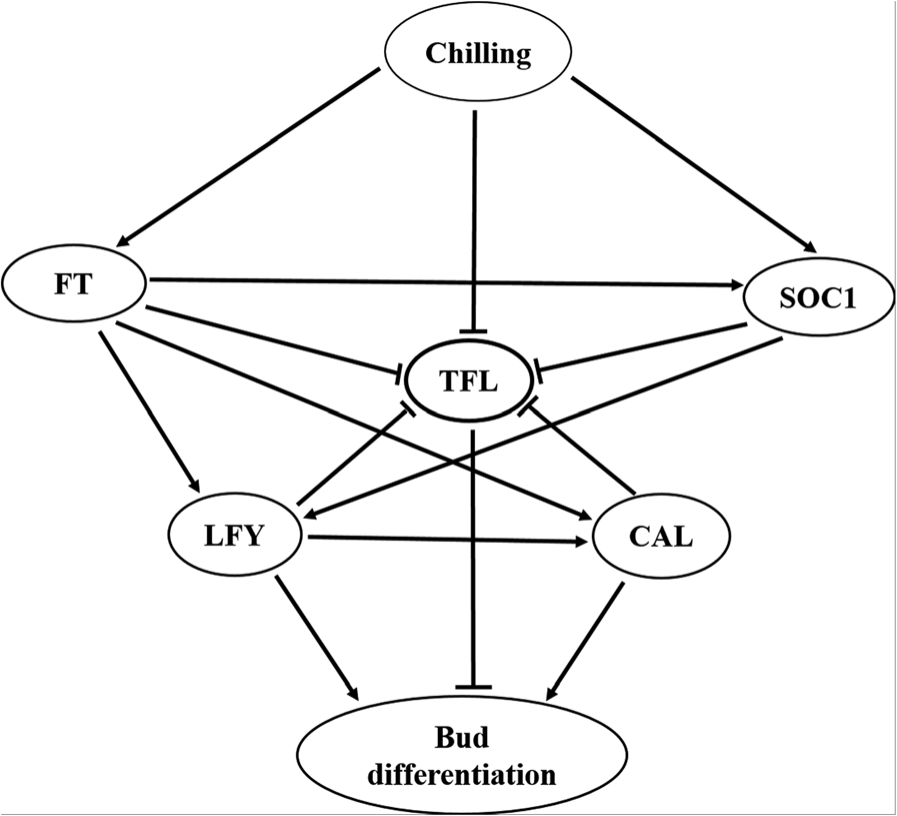
A simplified model of pathway controlling walnut flowering time based on Arabidopsis flowering pathway. The scheme shows the known genes involved in flowering regulation and the interactions between them and chilling requirement. *FT* and *SOC1* overexpress and encode proteins that activate the floral meristem identity genes such as *LFY* and *CAL* which convert the vegetative meristem to floral fate. *TFL1* as a flowering inhibitor gene has overexpression during the bud dormancy and its expression was decreased by meeting chilling requirement

The relationship between expression of genes herein was determined according to the Spearman correlation coefficient (GenEx software). Based on the results, the highest correlation was observed between *CAL* and *LFY* genes (r = 0.96). The *SOC1* gene correlated strongly with the *CAL* gene (r = 0.86) and the *LFY* gene (r = 0.86). On the other hand, the *TFL1* gene showed a negative correlation with the other genes (Table 1). Figure 2 shows the heat map and cluster analysis of the genes expressed in the terminal buds of the Persian walnut trees. Accordingly, the *TFL1* gene was classified in a separate group, which is due to its inhibitory effect on the flowering of walnut. The *CAL* and *SOC1* genes were responsible for flowering, hence they placed in one subgroup, whereas the two *LFY* and *FT* genes were clustered in another subgroup (Fig. 2). Figure 3 shows a proposed overview of the relationships among flowering genes involved in the flowering-time pathways of walnut based on Arabidopsis flowering pathway. By evaluating the interactions between the genes involved in flowering, it can be concluded that the end of winter (mid-March) is a time when the *TFL1* gene shows lower levels of expression, while higher levels of expression by the *FT* and *SOC1* genes were observed. Based on previous studies, it was expected that an increase in the expression of the *FT* gene would stimulate the expression of downstream genes involved in flowering. This increases the expression of *LFY* and *CAL* genes, whereby the differentiation of flower buds ensues.

**Table 1:** Spearman correlation between the flowering genes expression in walnut buds

	*FT*	*SOC1*	*CAL*	*LFY*	*TFL1*
*SOC1*	0.624	1			
*CAL*	0.442	0.865**	1		
*LFY*	0.447	0.858**	0.960**	1	
*TFL1*	−0.633	−0.786	−0.678	− 0.737	1

**Fig. 4:**
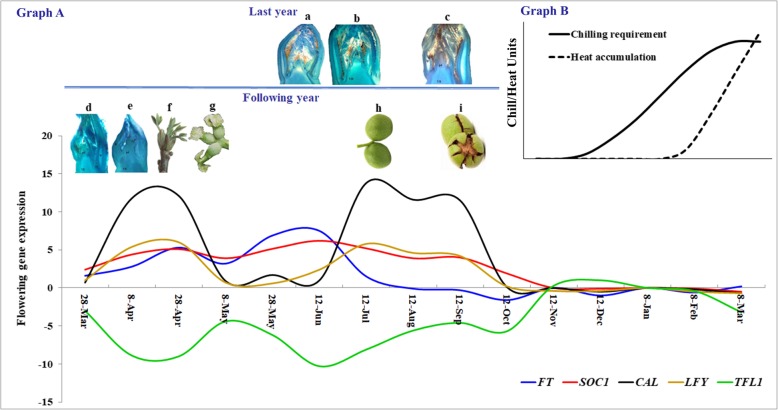
A general pattern of flowering genes expression and the date of different stages of flower development in walnut; flower induction (**a**, **b**) and initiation (**c**) occurred in the summer of last year. Flower differentiation (**d, e**) and anthesis (**f**, **g**) were observed in late March to early May of the same year. Harvest date (**i**) was from mid to late September. The graph C shows chilling and heat requirement of walnut trees cv. *Chandler*. An adequate winter chill and subsequent heat accumulation increase expression of flowering gene and release dormancy

Based on our results, after flowering, there was a decrease in the expression of the *FT* gene, followed by a decrease in the amounts of other flowering stimulators, whereas an increase in the expression level of *TFL1* gene was observed. However, this trend was completely reversed after a period of about one month, which was associated with an increase in the expression of *FT* and a reduction in the expression of *TFL1*. In early June, the expression of *FT* and *SOC1* genes increased, whereas the expression of the *TFL1* gene decreased. This balance caused the onset of flowering in buds, thereby preparing them for the following year. Following the increase in the expression of the *FT* gene, the expression level of *LFY* and *CAL* genes also increased, thereby leading to the flower induction in late summer. As autumn began, the expression of genes involved in flowering decreased, while the expression of *TFL1* increased. This trend continued throughout autumn, which was parallel to meeting the chilling requirement of buds. During autumn and winter, as the *TFL1* gene maintained a high level of expression, the flowering buds of walnut remained dormant. Ultimately, as the chilling requirement was met, the *TFL1* gene was expressed at a lower level, thereby making the buds ready for differentiation (Fig. 4).

## Discussion

Flowering is a complex event regulated by interactions between external and internal signals. The external flowering signals such as low temperature and light stimulate a network of internal transcription regulators that control flowering [23]. Our knowledge about the time of the different reproductive development stages is very important to ensure successful production and improve orchard management [24]. Previous studies indicated that walnut flower induction occurs a few weeks after the full bloom stage [5, 25–27]. Also, they state that flower initiation is in the late summer [24, 28]. Our results showed that walnut flower development like other fruit trees is divided into four stages including flower induction, initiation, differentiation and anthesis [5]. Walnut flower induction and initiation in the studied area (Shahmirzad, Semnan, Iran) occurred in early June and late summer, respectively. In general, walnut flower development (from induction to anthesis) lasts from June to May of the following year [29].

Flowering like other physiological process regulated by a large and complex gene networks. Certainly, studying the complete genome is necessary to gain a comprehensive understanding of the gene network involved in flowering. Utilizing powerful genetic tools based on next generation sequencing are an effective strategy for studying complex gene networks in flowering [19, 20]. Nevertheless, investigating the major genes (like *FT*, *SOC1*, *TFL1*, *CAL*, etc.) involved in flower development of plants can also improve our knowledge on the molecular mechanisms of flowering, especially in plants that have not been studied so far. The role of the *FT* gene in regulation of flowering has been identified in numerous species [19, 30–32]. Previous studies showed that *FT* gene promotes the transition to flowering and reproductive development [33, 34]. The results of the current study showed that the expression of the *FT* gene increased in three stages including before breaking of bud dormancy, from late March to late April and from late May to mid-June. Comparing the results of anatomical study of walnut flower development in Shahmirzad condition with the results of *FT* gene expression showed that *FT* gene was expressed slightly before flower differentiation. Also, the expression of *FT* gene was increased before budbreak and flower induction [33, 35, 36]. Based on our results, *FT* gene was found to be suppressed during bud dormancy in winter which indicated the role of *FT* gene in interacting with other genes in bud dormancy. Previous studies reported that *FT* plays an important role in winter dormancy especially eco-dormancy in plants such as *Populus* sp. [37, 38] and grape [39].

The results showed that the expression of *SOC1* increased during flowering and flowering induction. The expression pattern was significant at the end of bud dormancy. Lee et al. [40] reported that *SOC1* expression directly correlates with LEAFY expression and regulates the induction of flowering in flower development process. In *Arabidopsis*, the *SOC1* gene from the MADS-box has been proven to play an important role in integrating the flowering pathways. These genes are critical for determining the exact flowering time and supporting the maximum fertility. Furthermore, *SOC1* as a AGAMOUS-LIKE 20 or AGL20, independently and by the aid of two other groups, is reported as a positive flowering regulator [41, 42]. The *FT* and *SOC1* are likely representing the integration points of flowering pathways that enhance the expression of floral meristem identity genes [42–44]. A study on apple trees showed that the transcription of *MdSOC1* was activated when the flower was induced [25]. In Arabidopsis, *FT* activates the expression of *SOC1* [45]. According to the results of this study, the *FT* gene in the walnut is likely responsible for the activation of *SOC1*. Increasing the expression of the *SOC1* gene at the end of dormancy may be due to an increase in expression of the *FT* or it may be independently involved in dormancy release. This conclusion is based on some results in Arabidopsis which indicated that the *SOC1* gene is involved in the transduction of chilling signals [46] .

The *CAL* gene is a transcription factor that probably requlates the flora meristem identity genes in coordination with the *APETALA1*, *FRUITFULL* and *LEAFY* genes. This gene is the orthologous gene of *AP1* and the MADS-box genes. Our results indicated that the expression of *CAL* significantly increased at the bud break and flowering time. It might be due to the effects of this gene on the identification of flower organs in nuts. Ferrandiz et al. [47] determined that *FUL*, *AP1* and *CAL* control the formation of inflorescences by affecting the expression of *LFY* and *TFL1*, as well as their relative activity. The *LFY* protein directly activates the *AP1* protein and its homologous protein (*CAL*) in the flowers meristem [48, 49]. *AP1* B (Homologous *CAL* gene) is activated via *FT* [50]. The *CAL* and *FT* suppress the expression of *TFL1* in meristem.

TERMINAL FLOWER1 (*TFL1*) is a homologous protein to *FT* but has a different function in Arabidopsis [51, 52]. Increasing the expression of *AP1* in Arabidopsis decreased the expression of *TFL1*, indicating the activity of *AP1* as a suppressor of *TFL1* [53, 54]. Our results on walnuts also showed that there was a negative correlation between the *CAL* and *TFL1* gene. In other words, the *CAL* acts as a *TFL1* suppressor in the walnut. Given that *LFY*, *AP1*, and *CAL* are transcription factors, they are possibly directly interacting with cis-elements in the *TFL1* promoter [55]. Many studies on *LFY* have been conducted as a key control gene for flowering. In other words, the effective expression of *LFY* for the conversion of vegetative buds into the flowering ones is necessary, which indicates that this gene is a critical factor for the identification of flowers meristem [56, 57]. The *LFY* protein directly activates the *AP1* protein and its homologous *CAL* in the meristems [48]. He et al. [58] identified the *JrLFY* gene from Persian walnut. This gene consists of three exons and two introns. The sequencing of this gene is expected to form a polypeptide with 385 amino acids and has a conserved sequence within its C-terminal. They reported that the sequence of *LFY* protein in walnut is very similar to the one found in chestnut and hickory. The initiation of flowering and the transformation of the identity of floral meristem in Arabidopsis is largely dependent on the endogenous and environmental stimuli that ultimately cause the gene expression of *LFY* (*LEAFY*) and flowering conditions [59, 60]. The results of the present study indicated that the *LFY* expression increased before flowering and flowering time in walnut, and at other times, no significant levels of its expression were observed. Expression analysis of *FT*, *CAL*, *SOC1*, *LFY* and *TFL1* showed that at the end of the dormancy, the *TFL1* expression decreased while the *FT* and *SOC1* expression increased. At the end of the dormancy, the expression of the *FT* increased, which triggered the activation of the *SOC1* [45], that consequently led to expression of downstream genes, such as *LFY* and *CAL*. The expression of these genes significantly increased during the flower bud differentiation, which led to the flowering of walnut. After flowering, the expression *FT* and other flowering stimuli genes were decreased, but the expression of *TFL1* increased. In the following, the expression of the *TFL1* was reduced by increasing the expression of *FT* and other flowering stimuli genes which led to the flowering induction in walnut [42–44].

## Conclusion

*FT* gene is a primary stimulus gene in walnut flowering. The expression of *FT* gene not only increases in flower anthesis phase, but also increases in flower induction phase. *SOC1* is similar to *FT* showed overexpression in flower induction and anthesis stages. In contrast to *FT* gene, *SOC1* gene had a high level of expression in other times throughout growth season. The *LFY* and *CAL* genes are floral meristem identity genes which are activated by upstream genes such as *FT* and *SOC1* genes. *TFL1* is a flowering inhibitor gene in walnut which is overexpressed during the bud dormancy stage and chilling requirement reduces *TFL1* gene expression and overcome bud dormancy. In general, the interaction of flowering promoting genes such as *FT* and *SOC1* with flowering inhibiting genes including the *TFL1* gene modulate flowering transition and development including flower induction, initiation, differentiation and anthesis as well as bud dormancy of walnut trees. The present study provides an initial insight on the timing of the different stages of flower development in walnut and some major genes involved in this process. Definitely, a wide range of genes and gene networks as well as various pathways involved in walnut flowering which can be considered in the future. Based on the obtained results from gene expression as well as anatomical and phenological studies, the exact time of flower and fruit development stages of walnut from flowering to harvesting time was determined. Due to these results, fertilizer, irrigation and other orchard management factors can be adjusted.

## Methods

### Plant materials

This study was carried out in Shahmirzad Agro-Industry Company in Shahmirzad, Semnan Province, Iran. Shahmirzad (Latitude: 35.7729° N, Longitude: 53.3277° E, Altitude: ≈2050 m) which is located in a mountainous region on the southern slopes of the Alborz mountains, has a temperate climate with cool summers and cold winters. The plant materials were 15-years-old own-rooted Persian walnut trees (*Juglans regia* L. cv. Chandler) planted in a density of 5 m × 7 m. The studied walnut trees were produced by a Tissue Culture Laboratory namely RANA Agro-Industry Corporation, Iran. The most authentic material of ‘Chandler’ exists at University of California, Davis and does not have any voucher specimen and deposition number.

### Determining the time of flower induction, initiation and differentiation

Before studying the molecular mechanism of flower development, it was necessary to determine the time of different flower development stage (induction, initiation and differentiation) under the climate condition of Shahmirzad, Semnan. For this purpose, the anatomy of female flower bud was studied in three consecutive years (2014–2016). The buds were sampled throughout the year with 14-day intervals and were fixed overnight in fixation solution (including 10% formaldehyde + 5% acetic acid + 50% ethanol). The buds were cut vertically with a sharp razor and were stained with methylene blue and carmine [61]. The microscopy technique (Nikon Eclipse E200, Japan) was used for the determination of flower development stage.

### Assessment of chilling and heat requirement

Assessment of chilling and heat requirement of the studied walnut trees (*Juglans regia* L. cv. Chandler) was not one of the main objectives of this study. But, to better understand the expression patterns of the studied genes and to determine the precise time to meet chilling and heat requirement as well as release of bud dormancy, the chilling and heat requirement of walnut trees (cv. Chandler) were assessed. In the first step, we used one-year-old twigs with a terminal bud and catkin to determine chilling and heat requirement based on Utah and Growing Degree Hours (GDH°) models, respectively [62]. Six chilling treatments (650, 800, 950, 1100, 1250 and 1400 Utah Chilling Unit (CU)) and three replications were considered [3]. The results showed that the chilling requirement of terminal bud and catkin were 950–1100 and 800–950 CU, respectively. For evaluation heat accumulation, the studied twigs which received 950–1100 chilling unit, were placed in the greenhouse condition with a natural photoperiod and temperature between 16 and 23 °C. GDH° is considered as a degree upper threshold temperature for one hour. The heat requirement of walnut tree cv. Chandler was 11,832 GDH for terminal buds and 12,180 GDH for catkins.

In order to ensure the results of laboratory studies, chilling requirement and heat accumulation of the studied walnut trees were also evaluated based on Utah and Growing Degree Hours (GDH°) models, respectively [3]. Given that the phenological data of the studied walnut trees were available for 5 consecutive years (2013–2017), the 5-years meteorological data were used to evaluate chilling requirement and heat accumulation. Phenological data were evaluated based on IPGRI descriptor. CU and GDH were gathered from the IRIMO (Iran Meteorological Organization) website (http://www.irimo.ir) for the nearest synoptic station to the studied orchard. The data obtained from this section confirm the results of the laboratory study.

### RNA extraction and cDNA synthesis

In order to study the pattern of gene expression, 12 walnut trees cv. Chandler (3 replications and 4 trees in each replication) with the same age, trunk diameter and growth condition were selected. Total RNA was extracted from flowering buds throughout two consecutive years with 14-day intervals when the samples were prepared for anatomical study (2016–2017). RNA extraction was conducted using phenol saturated method described by Ghawana et al. (2011). To prepare extraction buffer, saturated phenol was added to sodium dodecyl sulphate (0.1% w/v), sodium acetate (0.32 M w/v) and EDTA (0.01 M). Approximately 0.1 g of bud sample was homogenized using a mortar and pestle to a fine powder in liquid nitrogen. A volume of 900 μl of extraction buffer, 100 μl of PVP solution and 5 μl of β-mercaptoethanol was added rapidly to the prepared powder and then was shaken vigorously. Then, 800 μl of DEPC-treated water was added and mixed by homogenizing for 5 min at room temperature. Chloroform (200 μl) was added to each tube and vortexed briefly (< 10 s) and left for 10 min at room temperature.

The prepared samples were centrifuged at 13,000 rpm for 10 min at 4 °C and the upper aqueous phase was transferred into fresh tubes. Then, 600 μl of isopropanol was added to tubes and vortexed briefly (< 10 s) and left for 10 min at room temperature. After that, the tubes were centrifuged at 13,000 rpm for 10 min at 4 °C and the supernatants were discarded. RNA pellet was washed with 70% ethanol and dissolved in 20 to 50 μl of DEPC-treated water. Quality of RNA was evaluated using 1% agarose gel. Also, purity and concentration of RNA was assessed by determining the absorbance of the sample at 260 and 280 nm using a spectrophotometer (PerkinElmer, Lambda 25, USA). Before cDNA synthesis, RNA was treated with RQ1 RNase-free DNase (Jena Bioscience, Germany). The first strand cDNA was synthesized by Easy cDNA synthesis kit (Pars Tous Biotechnology, Iran). PCR was performed in a 20 μl reaction containing 2 μl of cDNA, 0.6 mM dNTP, 1 μl of control primer, 1X reaction buffer, 0.7 mM MgCl2 and 1.5 U of Taq DNA polymerase.

### Primer design and gene-expression analysis by real-time PCR

The candidate genes in the current study were *FT*, *CAL*, *SOC1*, *LFY* and *TFL1*. The sequence of *CAL* and *LFY* genes in *Juglans regia* L. is available at NCBI website. The sequence of *FT* (*Malus domestica*; GenBank ID: DQ535887), *SOC1* (*Vitis vinifera*; GenBank ID: GU133633) and *TFL1* (*Malus domestica*; GenBank ID: NM_001293958) genes was used for a BLAST search in the walnut genome (https://www.ncbi.nlm.nih.gov/genome/17683). The sequences with the highest homology with the query sequences were used as templates to design primers. Therefore, the specific primers were designed for *FT*, *CAL*, *SOC1*, *LFY* and *TFL1* genes using Vector NTI and Oligo 3 software (Table 2). The purity and specificity of the designed primers were tested by PCR (Additional file 1).

**Table 2:** Annotation, sequences and accession numbers of primers used for gene isolation and quantitative real-time PCR analyzes

Annotation	Accession	5′ - primer- 3′
*FT-F*	DQ535887	GGTTGATGTAGGCGTTGATGAC CCGCTGTTGTTGCTGGAATATC
*FT-R*		
*CAL-F*	XM_018962972	CTGTGTGATGCTGAGGTCGG GTGACTAGCTTCGCCTCTGC
*CAL-R*		
*LFY-F*	JF520778	CTTCCATCACTACGAGCAGAGC CTCCAGCCTTCTTCGCATACC
*LFY-R*		
*SOC1-F*	GU133633	TCTTTGTGATGCCGAGGTTG TTGTTGATGGGTTGAGCGTC
*SOC1-R*		
*TFL1-F*	NM_001293958	TCACACTGGTCGAGACTGACC ACCTCCCTTCCTAATGTGGCTTC
*TFL1-R*		
*ACTIN-F*	XM_019003276	GGATGAGCAAGGAGATTACAGC TTGCGATCCACATCTGTTGG
*ACTIN-R*		

To study gene expression, real-time PCR was conducted using the LightCycler® 96 (Roche, Germany) with the SYBR Green real-time PCR Master Mix. Real-time quantification was performed in a reaction mix containing 2 μl cDNA, 0.2 μM gene-specific forward and reverse primers, 1X SYBER Green master mix in a final 20 μl reaction. Walnut actin gene (forward primer sequence: 5′- GGATGAGCAAGGAGATTACAGC-3′, and reverse primer sequence: 5′- TTGCGATCCACATCTGTTGG-3′) was used as the normalizer (Table 2). Real- time PCR cycling conditions were for 40 cycles of 15 s at 95 °C, 30s at the annealing temperature, 30s at the extension at 72 °C with 5 min at 72 °C for final extension.

### Statistical analysis

The data obtained from real-time PCR were analyzed by GenEx software. Samples taken at the beginning of the year (8th of January) were considered as the reference standard and the fold change gene expression were compared to this time.

## Supplementary information

**Additional file 1.** Agarose gel electrophoresis of PCR products of the designed primer involved in flowering including FT (≈130 bp), CAL (≈170 bp), TFL1 (≈120 bp), SOC1 (≈130 bp), LFY (≈125 bp) and Actin (≈140 bp) genes.

**Additional file 2.** Different flowering and fruit development stages of walnut during in three consecutive years (2014–2016) (Blue line: last year; Green line: following year).

**Additional file 3 **Fold changes of *FT* (a), *SOC1* (b), *CAL* (c), *LFY* (d) and *TFL1* (e) gene expression identified by quantitative RT-PCR analysis.

**Additional file 4.** The raw data obtained from real-time PCR and raw data to determine chilling requirement.

## Data Availability

All data analyzed during this study are included in this published article and its supplementary information files.

## Abbreviations

FT: FLOWERING LOCUS T
SOC1: SUPRESSOR OF OVEREXPRESSION OF CONSTANS 1
CAL: CAULIFLOWER
LFY: LEAFY
TFL1: TERMINAL FLOWER 1
