# Supplementary material for: Flowering in Persian walnut: patterns of gene expression during flower development
Source: BMC Plant Biol. 2020 Apr 3;20:136. doi: 10.1186/s12870-020-02372-w (PMC7118962; doi:10.1186/s12870-020-02372-w)
Supplement: Supplementary file 1 — Additional file 1. Agarose gel electrophoresis of PCR products of the designed primer involved in flowering including FT (≈130 bp), CAL (≈170 bp), TFL1 (≈120 bp), SOC1 (≈130 bp), LFY (≈125 bp) and Actin (≈140 bp) genes. [file 12870_2020_2372_MOESM1_ESM.docx]

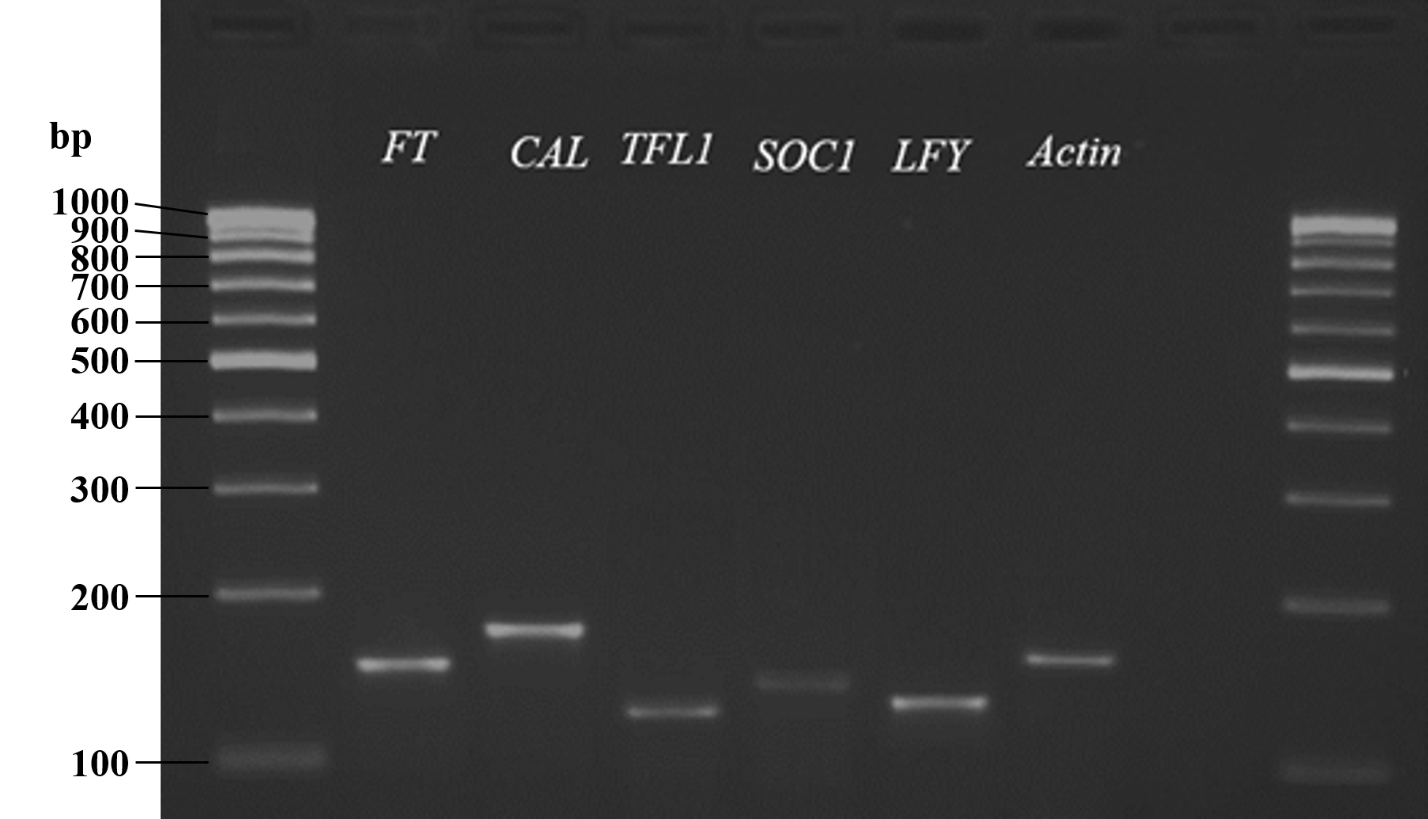


**Additional file 1.** Agarose gel electrophoresis of PCR products of the designed primer involved in flowering including *FT* (≈130 bp), *CAL* (≈170 bp), *TFL1* (≈120 bp), *SOC1* (≈130 bp), *LFY* (≈125 bp) and *Actin* (≈140 bp) genes
