# Supplementary material for: Flowering in Persian walnut: patterns of gene expression during flower development
Source: BMC Plant Biol. 2020 Apr 3;20:136. doi: 10.1186/s12870-020-02372-w (PMC7118962; doi:10.1186/s12870-020-02372-w)
Supplement: Supplementary file 2 — Additional file 2. Different flowering and fruit development stages of walnut during in three consecutive years (2014–2016) (Blue line: last year; Green line: following year). [file 12870_2020_2372_MOESM2_ESM.docx]

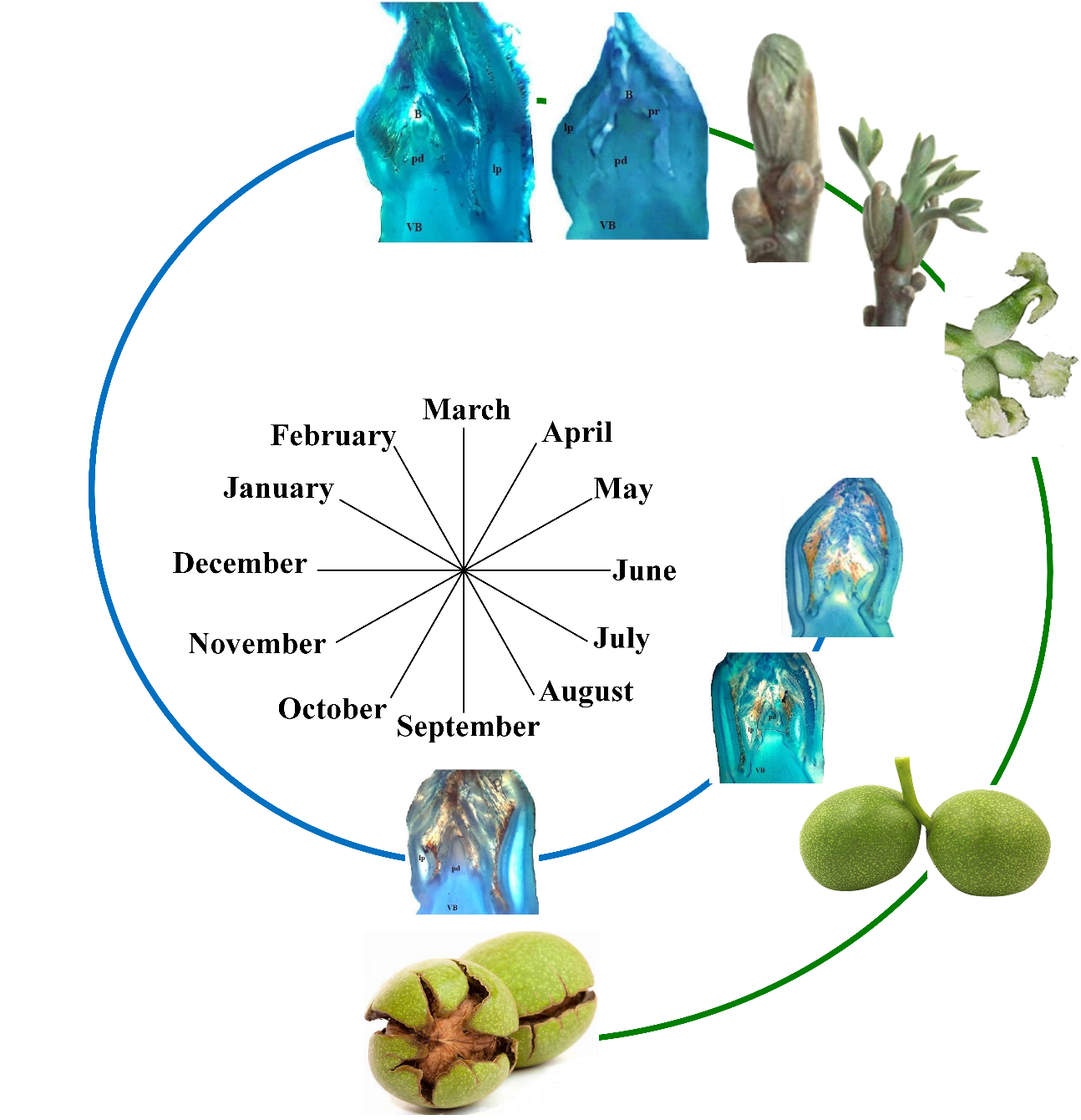


**Additional file 2.** Different flowering and fruit development stages of walnut during in three consecutive years (2014-2016) (Blue line: last year; Green line: following year)
