# Supplementary material for: Flowering in Persian walnut: patterns of gene expression during flower development
Source: BMC Plant Biol. 2020 Apr 3;20:136. doi: 10.1186/s12870-020-02372-w (PMC7118962; doi:10.1186/s12870-020-02372-w)
Supplement: Supplementary file 3 — Additional file 3 Fold changes of FT (a), SOC1 (b), CAL (c), LFY (d) and TFL1 (e) gene expression identified by quantitative RT-PCR analysis. [file 12870_2020_2372_MOESM3_ESM.docx]

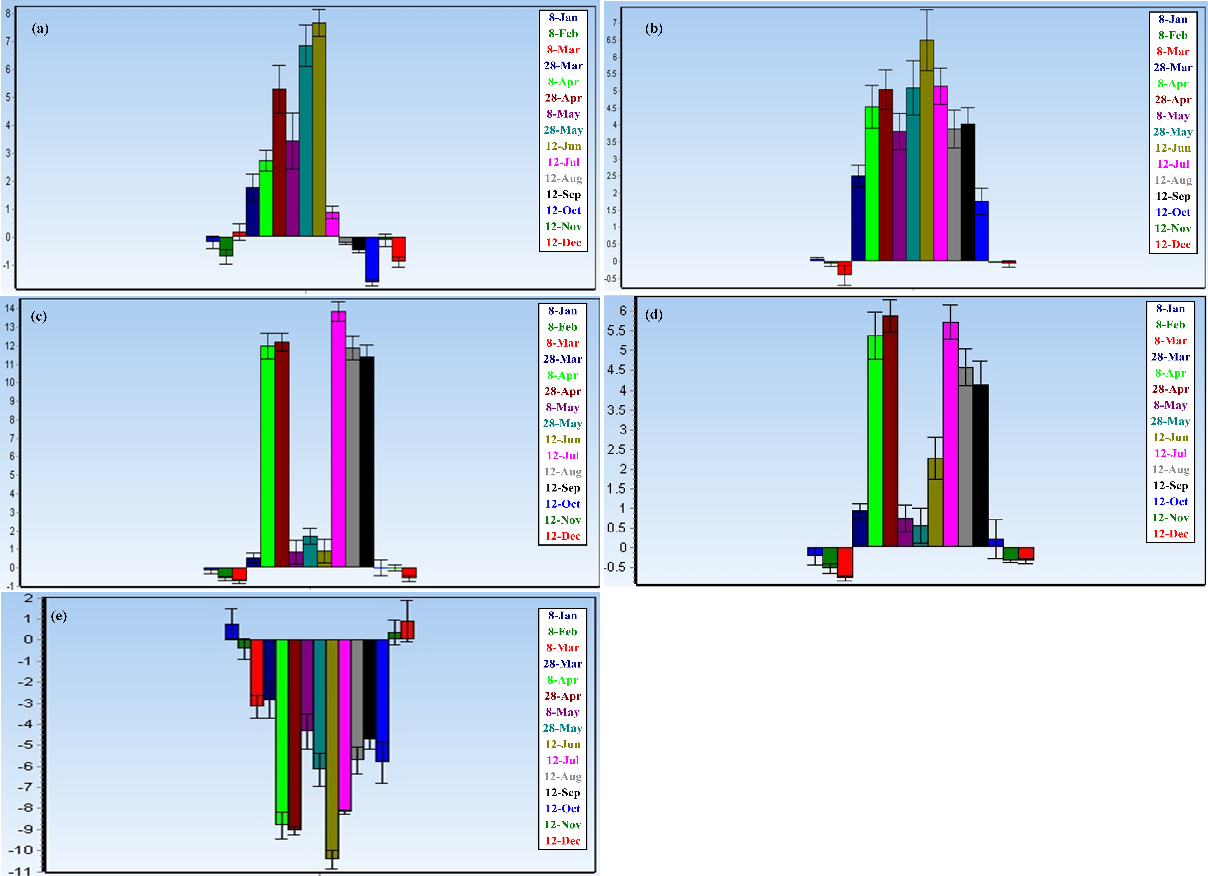


**Additional file 3.** Fold changes of *FT* (a), *SOC1* (b), *CAL* (c), *LFY* (d) and *TFL1* (e) gene expression identified by quantitative RT-PCR analysis
